# “What about that violence?” Transforming the discourse on violence in mental health research and services

**DOI:** 10.3389/fpsyt.2026.1905808

**Published:** 2026-08-20

**Authors:** Marichelle Leclair, Ashley J. Lemieux, Anne G. Crocker, Audrey-Anne Dumais Michaud, Daniela Perrottet, Luc Vigneault, Carolle Brabant, Suzanne Péloquin, Laurence Roy

**Affiliations:** 1 Department of Psychoeducation and Psychology, Université du Québec en Outaouais (UQO), Gatineau, QC, Canada; 2 Institut National de Psychiatrie Légale Philippe-Pinel, Montréal, QC, Canada; 3 Department of Sexology, Université du Québec à Montréal (UQÀM), Montréal, QC, Canada; 4 Department of Psychiatry and Addictology, School of Criminology, Université de Montréal, Montréal, QC, Canada; 5 School of Social Work and Criminology, Université Laval, Québec, QC, Canada; 6 Centre de Recherche en Santé durable VITAM, Québec, QC, Canada; 7 Centre de Recherche de Montréal sur les Inégalités Sociales, les Discriminations et les Pratiques Alternatives de Citoyenneté (CREMIS), Montréal, QC, Canada; 8 Department of Social and Preventative Medicine, Université Laval, Québec, QC, Canada; 9 Université Laval, Québec, Québec, QC, Canada; 10 CAP Santé Mentale, Québec, QC, Canada; 11 School of Readaptation, Université de Montréal, Montréal, QC, Canada

**Keywords:** epistemic violence, institutional violence, participatory research, reflexivity, vulnerability

## Abstract

Research on violence in mental health has long been dominated by behavioral science, risk assessment, and legal definitions of harm that conceptualize violence primarily as observable acts committed by individuals with mental illness. This orientation has produced a field capable of measuring and predicting such behavior, while largely failing to name the forms of violence experienced by people affected by mental illness, including institutional, structural, and epistemic harms. Grounded in reflexive engagement with RAP-Proches – a participatory action research project examining violence in the context of mental illness and family caregiving in Québec, Canada –, and drawing on peer-reviewed literature across psychiatry, psychology, public health, and social sciences, we seek to transform mental health research and services by proposing a reconceptualization of violence in mental health using Johan Galtung’s framework of personal, structural, and cultural violence. Galtung’s triangle reveals how these three forms are interconnected and mutually reinforcing: cultural violence legitimizes structural violence, which in turn shapes the conditions under which personal violence occurs. Put in dialogue with Miranda Fricker’s theory of epistemic injustice, this framework shows how the concentration of research on personal violence reproduces the conditions under which institutional and structural harm remains unnameable, leaving those harmed by institutions without the conceptual tools to name that harm as violence. We identify conditions for epistemic justice in research (including reciprocity, acknowledgment of trauma, and recognition) as concrete mechanisms for disrupting these dynamics.

“*When are we going to talk about the violence that mental health services perform on us? What about that violence? Are we going to at last discuss what we are doing here?*”

– co-researcher with lived experience as a person diagnosed with mental illness

“*I had planned to pick up my son at the hospital so that I could see him for the first time since his suicide attempt. They didn’t tell me he was leaving the unit to come to my home. When my son arrived at my place with visible scars on his neck – when I saw my son, my own son, in that state – I couldn’t open the door. I wanted to welcome him, but I was in distress. I said, ‘I can’t see you like this.’ He began banging on my door insistently. Was he violent? Yes. But his violence was a response to my inability to open my door to him, and my inability was the result of the psychiatric institution’s violence toward us.”*

– co-researcher with lived experience as a family member of a person diagnosed with mental illness

Both of these statements emerged during a virtual team meeting in the first months of a participatory action research project on the needs of family members experiencing situations of violence in the context of caring for a loved one with mental illness. I, the senior author of this text, was sitting in the open area where I work remotely from home, and the scene beyond the computer screen was filled with artifacts and objects belonging to my children. When a co-researcher shared stories of being deprived of her right to act as a loving, caring mother to her adult son with a mental illness by psychiatric institutions and their habitual ways of doing, I experienced what Minatti and Gass-Quintero (1) call “the epistemic value of vulnerability”. I closed my camera. Others on the call were visibly crying. This significant moment (2) in the participatory process allowed us to reformulate the research questions we had started with, and to see the field of mental health and violence from a different angle, thus effectively initiating a process of transformation.

Our team, based in Québec, Canada, obtained provincial funding for this research project in 2023. From the start, the project (RAP-Proches^1^) was framed as participatory, initiated through a partnership between a collaborative of support groups for families of individuals living with mental illness (Cap Santé Mentale) and a research team with expertise at the intersection of mental illness, mental health services, and violence. Its initial focus on interpersonal violence – more specifically, overt violence exerted by persons with mental illness towards family members – was grounded in a community-identified need: the lack of non-judicial resources and levers for families in the context of intra-familial violence. We had decided to encourage dialogue between multiple perspectives to allow for alignment with as many interested parties as possible. In hindsight, both the structure of the participatory process – closely aligned with the imperatives of the funding opportunity and the pace of academic grant writing, reflecting the neoliberal nature of contemporary academia (3) – and our initial conceptualization of violence produced harm for the co-researchers with lived experience of mental illness and for those with lived experience of caring for loved ones with mental illness. It was when the experience became personal – as reflected in the situation described in the opening paragraphs of this paper – (4) that transformation could start.

Two things changed at the same time, through that transformation. First, we widened what we understood as violence, beyond acts committed by one person against another. Second, we changed who, within the team, was recognized as able to say what counts as violence at all. The two went together – we could not have broadened the definition without first changing whose knowledge we treated as credible. This created a real difficulty. We had begun by treating one form of violence (that of a person with mental illness toward a family member) as a problem to study on its own. But what the co-researchers with lived experience showed us is that this form of violence could not be understood apart from the institutional violence surrounding it. Our challenge was therefore not to separate interpersonal from institutional violence, but rather to stop conceptualizing them as distinct.

In this paper, we hope for (2) a broader transformation of how violence is understood and used in mental health research and services, moving away from the dominant, “risk^2^”-based discourses that treat violence primarily as an individual act. Specifically, our objectives are (1): to critically examine dominant conceptualizations of violence in mental health research (2); to propose a reconceptualization of violence in mental health grounded in Johan Galtung’s theoretical framework of personal, structural, and cultural violence; and (3) to examine how spaces of epistemic justice can disrupt the production of violence within research itself.

## Violence as “the act”: the dominant paradigm and its consequences

Violence is a concept that has been defined differently across disciplines, including law, sociology, psychology, and public health (5, 6). These definitions differ in their emphasis on intent, harm, thresholds of severity, and the forms of violence they seek to capture (5). In this section, we briefly review common definitions and conceptualizations of violence, and argue that, despite their diversity, most definitions converge around a remarkably narrow understanding of violence: namely, interpersonal, observable acts of harm, most often attributed to individual actors.

### Violence as individual behavior

Research in mental health and violence has been largely anchored in approaches derived from the behavioral sciences (7). Within these traditions, violence is predominantly conceptualized as an individual behavior that is interpersonal, observable, and attributable to a specific actor (5). It is most often defined in terms that align with legal conceptions of harm, including acts such as physical assault, threats, or behaviors likely to result in criminal sanctions.

Influential epidemiological and clinical studies have played a central role in this orientation. Early large-scale studies, such as the Epidemiological Catchment Area study (8), operationalized violence through self-reported acts of aggressive behavior (9, 10). Across these and subsequent studies, violence is rarely questioned as a conceptual construct; rather, it is operationalized through specific behaviors and events. Most consistent with these operationalizations is Hamby’s definition, according to which “violence is a behavior that is (a) intentional, (b) unwanted, (c) nonessential, and (d) harmful” (5).

This orientation has been further reinforced by the development of violence risk assessment tools, which have become central to research and practice in mental health (11). Over the past decades, more than 200 violence risk assessment and management tools (12) have been developed. Within this framework, violence is not only conceptualized as an individual behavior but also as a measurable outcome that can be predicted, managed and controlled. The emphasis on prediction and risk management has contributed to a paradigm in which violence is primarily understood as a problem of individual propensity. Ethnographic research conducted within the various social institutions where risk of violence is assessed, managed, monitored and reformed illustrates how public opinion, legal and medical processes legitimize the deployment of force (e.g. coercive practices), by conflating notions of risk and dangerousness and framing both as inherently individual attributes (13–15).

Although more recent work has sought to broaden this focus – for instance by including victimization or self-directed violence (16) – these efforts have not fundamentally altered the dominant orientation of the field. Across contexts, violence is operationalized through behavioral, clinical, or legal indicators that prioritize observable acts attributable to individuals living with mental illness. This orientation is closely tied to the methodological and institutional demands of clinical, forensic, and epidemiological research, which require standardized, measurable outcomes (17, 18). However, it also has important implications for what is recognized (and not recognized) as violence.

### What the public health framework adds – and misses

Public health frameworks expand conceptualizations of violence beyond discrete acts of physical aggression. The World Health Organization (WHO) defines violence as “the intentional use of physical force or power, threatened or actual, against oneself, another person, or against a group or community, that either results in or has a high likelihood of resulting in injury, death, psychological harm, maldevelopment or deprivation” (19). The WHO further proposes a typology distinguishing self-directed, interpersonal, and collective (e.g., genocide) forms of violence, each of which may manifest through physical, sexual, psychological harm, or deprivation.

This broader conceptualization has been particularly influential in positioning violence as a global public health issue (6). By including notions of power, neglect, and deprivation, the WHO framework opens the possibility of recognizing forms of violence that are not reducible to interpersonal behaviors of aggression. Yet even this expanded framework tends to locate violence in the actions of identifiable actors or groups; it stops short of accounting for forms of harm that originate in institutions, professional practices, and systems of knowledge. While the WHO definition is widely cited in the literature – typically in introductory sections to establish scope – empirical studies consistently operationalize violence through narrower behavioral measures (7).

## Violence beyond the act

### Galtung’s triangle

The limitations identified in dominant conceptualizations of violence call for a framework capable of accounting for forms of harm that extend beyond observable, interpersonal acts. Johan Galtung’s work offers such a framework. Originally developed within peace and conflict studies, Galtung’s conceptualization of violence provides a broader and more structurally grounded understanding that is particularly useful for re-examining violence in mental health.

Galtung defines violence as “the cause of the difference between the potential and the actual, between what could have been and what is” (20, p. 168). In other words, violence is present whenever people are kept from reaching their full potential due to avoidable factors. This definition marks a significant departure from dominant approaches in behavioral sciences in general and mental health research in particular: violence is no longer limited to discrete acts, nor does it require a clearly identifiable perpetrator or intent. Rather, it encompasses a wide range of conditions that produce harm, whether directly or indirectly, intentionally or not.

To account for this complexity, Galtung proposes a typology composed of three interrelated forms of violence: personal (or direct), structural, and cultural. Personal violence refers to observable acts of harm inflicted by identifiable actors, including physical, psychological, or emotional injury. Structural violence refers to forms of harm embedded in social and institutional systems that constrain individuals’ ability to meet their needs and reach their full potential. As Galtung notes, “violence is built into the structure and shows up as unequal power and consequently as unequal life chances.” (20). Unlike personal violence, structural violence does not occur through identifiable events, but through enduring processes, including rules, procedures, practices, and policies. Cultural violence, introduced later (21), refers to the symbolic, ideological, and epistemological systems that legitimize structural violence. It operates through dominant norms, beliefs, and discourses that make certain forms of harm acceptable, justified or inevitable. As Galtung argues, cultural violence makes “structural violence look, even feel, right – or at least not wrong” (21).

Rather than operating independently, each form of violence legitimizes and sustains the other. Cultural violence legitimizes structural violence, which in turn shapes the conditions under which personal violence may occur. At the same time, acts of personal violence can reinforce the cultural narratives that justify structural harm. These forms of violence differ in their visibility: personal violence is episodic and easily observable, whereas structural and cultural violence operate as slower, less visible processes. As a result, empirical research tends to privilege the former, while the latter remain more difficult to capture and, consequently, less frequently studied.

### Applying the triangle to mental health research and services

**Figure 1:**
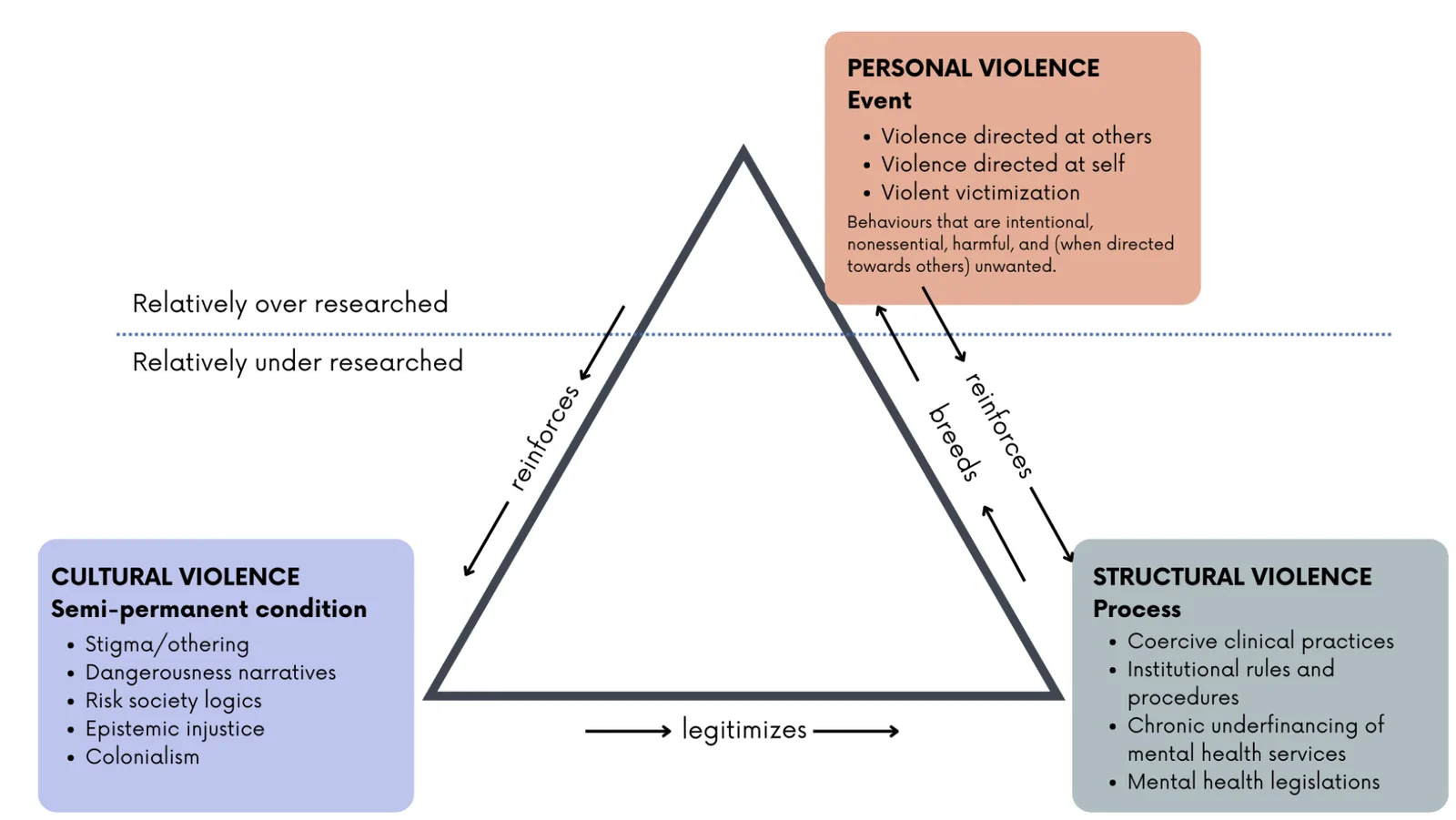
A typology of violence in mental health, adapted from Galtung (1990).

Applying Galtung’s framework to mental health allows us to account for forms of violence that are largely unexamined within dominant research paradigms (see **Figure 1**). While personal violence has been extensively studied – most often in the form of aggression or violent crimes committed by individuals with mental illness (e.g., Fazel et al., 2009; Whiting et al., 2021; Witt et al., 2013) – structural and cultural forms of violence remain comparatively underexamined (22).

In a mental health context, personal violence among people with mental illness can include violence directed at others, violence directed at self, and violent victimization (16). In relation to personal violence, we may rely on Hamby’s definition, according to which violence is a behavior that is intentional, nonessential, harmful, and – when directed towards others – unwanted (5).

Structural violence, by contrast, can be observed in the organization and delivery of mental health care (23), as well as in policies that shape access to housing, employment, care, citizenship, etc. Institutional practices such as involuntary admission, seclusion, restraint, and other practices that restrict autonomy and impede dignity often create harm, such as increased risk of suicide or feelings of dehumanization (24–27), even when implemented within legal and clinical frameworks. More broadly, systemic issues such as unequal access to services (28, 29), cultural unsafety (30), and structural stigma (31) contribute to shaping the life trajectories of individuals with mental illness, limiting opportunities for recovery and healing. These forms of structural violence are likely to operate differently across dimensions of race, gender, class, and diagnosis (e.g., substance use disorders) (15, 32). The situation described in the opening vignette can be understood as a form of structural violence. Structural violence creates the conditions, or “breeds” to use a Galtung-like terminology, personal violence by creating conditions of distress, frustration, and powerlessness that may give rise to acts of personal violence (33). In return, these acts are interpreted through risk-oriented frameworks, reinforcing perceptions of dangerousness and justifying further restrictive measures, tighter procedures, and increased surveillance.

Cultural violence legitimizes structural violence. In mental health, this includes dominant narratives that portray people with mental illness as dangerous or unpredictable, reinforcing the prioritization of risk management over other forms of care (34). Society’s focus on risk (35, 36) – which has become a defining feature of contemporary mental health governance (37) – further frames the prevention of rare but high-consequence events as a primary objective, often at the expense of the rights and liberties of many. In risk societies, Giddens argues, expanding scientific knowledge does not produce greater security; rather, it generates new uncertainties and an intensified preoccupation with controlling the future. This dynamic helps explain why the accumulation of risk assessment tools has not reduced “coercive” practices but expanded them (38, 39). The aspiration to predict and control future violence justifies restricting liberty in the present: it has been estimated that restricting the liberty of 26 individuals with mental illness identified as high risk is required to prevent a single violent offense, and over 2,500 to prevent one homicide (40). Yet, the corresponding benefit (that acting on these tools meaningfully reduces violence) remains largely untested (41–43), particularly among women (44).

Cultural violence also operates at the level of knowledge production. Post-positivist epistemologies remain dominant (17, 18), with models of evidence such as randomized controlled trials frequently treated as the gold standard for informing policy and practice. While these approaches offer important contributions, they privilege forms of knowledge that are quantifiable, generalizable, and predictive. In doing so, they tend to marginalize or exclude other forms of knowledge, including Indigenous epistemologies, relational ways of knowing, and the experiential knowledge of individuals and families (11). This hierarchy of knowledge narrows what counts as meaningful data, valid evidence, or effective intervention, and plays a central role in rendering certain forms of harm – particularly those embedded in relationships, institutions, and lived experience – less visible and less contestable.

## When knowledge does violence: epistemic injustice in mental health

Epistemic injustice can be understood as a specific mechanism through which cultural violence operates in mental health. Drawing on Fricker (45), we understand epistemic injustice as forms of oppression that manifest through the production, circulation, and recognition of knowledge (46). It is rooted in the marginalization or delegitimization of certain forms of knowledge and of groups who hold them, resulting in unequal access to meaning-making (45). These epistemic relations actively produce forms of ignorance by preventing knowledge circulation (47).

Fricker characterizes two forms of epistemic injustice: testimonial injustice – when a prejudice decreases the level of credibility awarded to a speaker – and hermeneutical injustice – when someone or a group is deprived of the necessary resources to understand and express their own experiences due to a system of knowledge dominated by hegemonic groups (45).

In the field of mental health, both forms of epistemic injustice are distinctly at work. Testimonial injustice occurs when people living with mental illness attempt to articulate experiences of harm (whether arising from coercive practices, institutional discrimination, or structural neglect) and find their accounts discredited, reframed as symptoms, or dismissed. The psychiatric diagnosis itself can function as what Fricker calls an “identity prejudice”, that is, a marker that systematically deflates the speaker’s credibility as a knower of their own experience. Hermeneutical injustice operates at a more structural level. When the dominant discourse on violence in mental health reserves the concept almost exclusively for acts committed by people with mental illness, it leaves those same individuals without the conceptual resources to name what is done *to* them as violence (15). As one person put in a participatory research project on violence: “The word ‘violence’ is so often used *about* us that it’s not a word we use easily. How can I say *I’m* a victim of violence when people always told me [ … ] that it’s us who are violent?” (4). This hermeneutical gap constitutes a form of violence in its own right. This dynamic is self-reinforcing: the more a group’s knowledge is dismissed, the less it participates in producing knowledge, and the more its experiences remain unnamed (45).

### Reflexivity and vulnerability against epistemic injustice

While participatory research has become increasingly popular in the field (48) and constitutes one way to respond to epistemic injustice (49), we argue that representation as participation, that is, simply having people with lived experience, family members and community members “at the table”, is not true participatory research. Responding to epistemic violence in mental health research requires researchers to adopt reflexive and vulnerable stances, as well as to recognize the potential for harm in research relationships (50). Reflexivity here means that researchers can use their own experiences as a conduit for understanding others’ perspectives or situations (51). Regarding vulnerability, research has traditionally conceptualized it as a characteristic of individuals and groups, including those living with mental illness. An alternative conceptualization understands vulnerability as a set of experiences and situations (1). Participatory research offers researchers an opportunity to experience vulnerability as an analytical tool: it allows them to use their own experiences of being affected by, or imagining, potential harm to broaden their understandings of violence. In the introduction, we highlighted how these experiences of researcher vulnerability opened up a new understanding of violence when we collectively imagined situations of institutional violence that would cause harm to our loved ones and ourselves.

These considerations can be further refined by attending to Skelton’s conditions for epistemic justice in research (4). Our own research project, RAP-Proches, offers a concrete illustration of how these conditions can be enacted in practice. The first is reciprocity: engaging with the research topic through personal experience is expected of everyone involved, regardless of status or role. In RAP-Proches, conversations on institutional violence deepened when clinicians and academic researchers (not only those with lived experience) shared personal stories about caregiving and navigating health systems. The second condition is acknowledging trauma and the risks involved in breaking silence. Pre-existing relationships between people with lived experience of mental illness, family members, and researchers created conditions in which sensitive issues such as institutional violence could be named early in the research process – a possibility that required building trust and respecting each person’s rhythm. The third condition is recognizing all participants as knowers with their own agency, who share co-ownership of the research process at each step. In RAP-Proches, this involved co-developing the research protocol, slowing down and extending the research timeline, as well as creating conditions to facilitate co-authorship (translating all the material from English to French, relying more heavily on dialogue than on co-writing). This condition was central to a shift in the conceptualization of violence. At the same time, the potential for co-ownership cannot be fully achieved in RAP-Proches as in many participatory research projects that operate within the imperatives of systems where academic researchers maintain unbalanced ownership over grant funding, publications, and dissemination activities (52). Finally, the fourth condition is fostering a sense of belonging through the collective. Within our group, this took shape as collaborations outside of the research context, such as a providing a joint testimony in front of the National Assembly of Québec as part of a public consultation on a bill aimed at enhancing oversight and follow-up mechanisms for individuals found not criminally responsible on account of mental disorder.

## Conclusion

The dominant paradigm around violence in mental health has long been rooted in behavioral science, risk assessment, and legal definitions of harm, treating violence primarily as an attribute or problem of the individual rather than a product of broader social or systemic factors. As researchers, we have been and are complicit in sustaining this narrow picture: our sustained involvement in predicting and managing violent behavior has produced a field that knows how to measure the violence of people with mental illness and yet struggles to even name the violence against them. The questions raised by co-researchers with lived experience at the outset of this paper reveal what the dominant paradigm has rendered unspeakable.

Galtung’s triangle (20, 21) disrupts this by making personal, structural, and cultural violence visible in their interconnection. Put in dialogue with Fricker’s (45) notion of epistemic injustice, it shows how the field’s disproportionate concentration on personal violence can leave people harmed by institutions through structural and cultural violence without the credibility and conceptual tools to name that harm as violence, thereby contributing to the legitimization of the very structural violence it fails to see. Our argument, then, is not only that violence in mental health has been too narrowly defined, we argue that this definition has, in turn, organized what can be known, whose suffering becomes legible and legitimate, and which harms are rendered visible or ignored within research, policy and practice.

Galtung was as interested in peace as he was in violence. For him, peace meant the absence of violence in all its forms (20). The transformation of research discourse toward epistemic justice is, in this sense, a form of peace. It is also a necessary condition for building mental health research capable of apprehending violence not only as an individual act, but as a social, institutional and epistemic relation. Such a shift invites researchers to move beyond the mere expansion of violence categories and toward a deeper reorientation of the questions we ask, the knowledge we recognize as credible, and the forms of harm we are prepared to confront. The question we are left with is, what would it mean for mental health research to foreground peace, rather than violence?

## Data Availability

The original contributions presented in the study are included in the article/supplementary material. Further inquiries can be directed to the corresponding author.

## Footnotes

1 A French word play on participatory action research (PAR, or RAP in French) and “proches”, which is a term frequently used for family and friends, and literally means ‘those close to us’.

2 We use “care”, “risk”, and “coercion” here as shorthand, aware that they are contested terms. Ethnographic work in medical anthropology shows that care and violence often coexist within the same practices (5,6).
